# Atherogenic dyslipidemia in metabolic syndrome and type 2 diabetes: therapeutic options beyond statins

**DOI:** 10.1186/1475-2840-5-20

**Published:** 2006-09-26

**Authors:** Alexander Tenenbaum, Enrique Z Fisman, Michael Motro, Yehuda Adler

**Affiliations:** 1 Cardiac Rehabilitation Institute, the Chaim Sheba Medical Center, 52621 Tel-Hashomer, Israel; 2 Cardiovascular Diabetology Research Foundation, 58484, Holon, Israel; 3 Sackler Faculty of Medicine, Tel-Aviv University, 69978 Ramat-Aviv, Israel

## Abstract

Lowering of low-density lipoprotein cholesterol with 3-hydroxy-3-methylglutaryl coenzyme A reductase inhibitors (statins) is clearly efficacious in the treatment and prevention of coronary artery disease. However, despite increasing use of statins, a significant number of coronary events still occur and many of such events take place in patients presenting with type 2 diabetes and metabolic syndrome. More and more attention is being paid now to combined atherogenic dyslipidemia which typically presents in patients with type 2 diabetes and metabolic syndrome. This mixed dyslipidemia (or "lipid quartet"): hypertriglyceridemia, low high-density lipoprotein cholesterol levels, a preponderance of small, dense low-density lipoprotein particles and an accumulation of cholesterol-rich remnant particles (e.g. high levels of apolipoprotein B) – emerged as the greatest "competitor" of low-density lipoprotein-cholesterol among lipid risk factors for cardiovascular disease. Most recent extensions of the fibrates trials (BIP – Bezafibrate Infarction Prevention study, HHS – Helsinki Heart Study, VAHIT – Veterans Affairs High-density lipoprotein cholesterol Intervention Trial and FIELD – Fenofibrate Intervention and Event Lowering in Diabetes) give further support to the hypothesis that patients with insulin-resistant syndromes such as diabetes and/or metabolic syndrome might be the ones to derive the most benefit from therapy with fibrates. However, different fibrates may have a somewhat different spectrum of effects. Other lipid-modifying strategies included using of niacin, ezetimibe, bile acid sequestrants and cholesteryl ester transfer protein inhibition. In addition, bezafibrate as pan-peroxisome proliferator activated receptor activator has clearly demonstrated beneficial pleiotropic effects related to glucose metabolism and insulin sensitivity. Because fibrates, niacin, ezetimibe and statins each regulate serum lipids by different mechanisms, combination therapy – selected on the basis of their safety and effectiveness – may offer particularly desirable benefits in patients with combined hyperlipidemia as compared with statins monotherapy.

## Review

Lowering of low-density lipoprotein (LDL) cholesterol with 3-hydroxy-3-methylglutaryl coenzyme A reductase inhibitors (statins) is clearly efficacious in the treatment and prevention of coronary artery disease (CAD) [1-8]. However, despite increasing use of statins, a significant number of coronary events still occur and many of such events take place in patients presenting with type 2 diabetes and metabolic syndrome. More and more attention is being paid now to combined atherogenic dyslipidemia which typically presents in patients with type 2 diabetes and metabolic syndrome [9]. This mixed dyslipidemia (or "lipid quartet"): hypertriglyceridemia, low HDL (high-density lipoprotein)-cholesterol levels, a preponderance of small, dense LDL particles and an accumulation of cholesterol-rich remnant particles (e.g. high levels of apolipoprotein B) – emerged as the greatest "competitor" of LDL-cholesterol among lipid risk factors for cardiovascular disease. The lifestyle changes recommended by the National Cholesterol Education Program (NCEP) Adult Treatment Panel (ATP) III for controlling dyslipidemia (i.e., elevated levels of triglycerides and decreased levels of HDL-cholesterol) in patients with metabolic syndrome or type 2 diabetes mellitus (DM) include (1) reduced intake of saturated fats and dietary cholesterol, (2) intake of dietary options to enhance lowering of low-density lipoprotein cholesterol, (3) weight control, and (4) increased physical activity. If lifestyle changes are not successful for individuals at high risk of developing CAD, or for those who currently have CAD, a CAD risk equivalent, or persistent atherogenic dyslipidemia, then pharmacotherapy may be necessary. Current therapeutic use of statins as monotherapy even in optimal doses and achieved target LDL- cholesterol reduction is still leaving many patients with mixed atherogenic dyslipidemia at high risk for coronary events. Targeting multiple lipid pathways can provide greater reductions in LDL-C as well as improvements in other lipid parameters. In the current article we briefly examine recent data regarding different lipid-lowering approaches (non-statin-based or combined strategies) in patients with mixed atherogenic dyslipidemia.

### Fibrates: new evidences from HHS, BIP extensions and FIELD

Fibrates have been used in clinical practice for more than four decades due to their ability substantially to decrease triglyceride levels, to increase HDL-cholesterol levels and in addition to reduce LDL-cholesterol moderately but significant [9].

Due to their beneficial effects on glucose and lipid metabolism, PPAR's alpha agonists (fibrates) are good potential candidates for reducing the risk of myocardial infarction (MI) in subjects with metabolic syndrome and diabetes [10-12]. Although less clinical intervention studies have been performed with fibrates than with statins, there are evidences indicating that fibrates may reduce risk of cardiovascular disease and particularly non-fatal MI [13-19]. Interestingly, reduction of cardiovascular disease with two of the fibric acid derivates – gemfibrozil and bezafibrate – was more pronounced in patients displaying baseline characteristics very similar to metabolic syndrome definitions [13,14,20].

There have been no direct head-to-head comparisons of a statin with a fibrate in any clinical endpoint trial. However, compared with statins, fibrates appear to more selectively target the therapeutic goals in obese individuals with features of insulin resistance and metabolic syndrome (i.e. with near-goal LDL-cholesterol and inappropriate HDL-cholesterol and triglyceride levels).

#### Gemfibrozil: confirmed long-term efficacy

The primary-prevention trial Helsinki Heart Study (HHS) showed that treatment with gemfibrozil led to a significant reduction in major cardiovascular events [13]. Regarding secondary prevention, in the VA-HIT study (Veterans Affairs High-density lipoprotein cholesterol Intervention Trial) – which included 30% of diabetic patients – gemfibrozil reduced the occurrence of major cardiovascular events by 22 % [14]. Similarly, reduction of cardiovascular disease with gemfibrozil was more pronounced in patients displaying above three of the features of metabolic syndrome [21,22].

The 18-year results from the Helsinki Heart Study shows that patients in the original gemfibrozil group had a 23% lower risk of CAD mortality compared with the original placebo group. But those in the highest tertile of both body-mass index and triglyceride level at baseline had the most dramatic risk reductions with gemfibrozil – 71% for CAD mortality and 33% for all-cause mortality [23].

These results are entirely consistent with the original positive results of HHS and are strongly supported by the findings of VA-HIT.

#### Fenofibrate: disappointing results of the FIELD

The recent Fenofibrate Intervention and Event Lowering in Diabetes (FIELD) study (24 investigated the effects of fenofibrate on cardiovascular events in type 2 diabetes patients. This was a multinational, randomized, double-blind, placebo-controlled trial in 9795 subjects aged 50 to 75 years of age with type 2 diabetes who were not prescribed statin therapy at study entry. The primary endpoint was coronary events (CAD death or nonfatal MI). The prespecified endpoint for subgroup analyses was cardiovascular events (cardiovascular death, MI, stroke, and coronary and carotid revascularization procedures). After 5 years, fenofibrate-treated patients had a nonsignificant 11% reduction in the incidence of the primary endpoint, nonfatal myocardial infarction, or CAD death (5.2% event rate for the fenofibrate group compared with 5.9% for the placebo group; *P *= 0.16). Fenofibrate treatment did, however, reduce the incidence of the broader total cardiovascular events endpoint (a prespecified secondary endpoint) by 11% (*P *= 0.035). Fenofibrate reduced the incidence of most other prespecified endpoints of macrovascular disease, including nonfatal MI events by 24% (*P *= 0.01), coronary revascularizations by 21% (*P *= 0.003), and all revascularizations by 20% (*P *= 0.001). Fenofibrate treatment had a particularly beneficial effect in patients that had no prior CAD. In this primary prevention population (78% of the total population), fenofibrate reduced the incidence of the primary endpoint (CAD events) by 25% (*P *= 0.014) and the incidence of total cardiovascular events by 19% (*P *= 0.004). In addition, fenofibrate unexpectedly showed statistically significant reductions in several endpoints, suggesting that microvascular benefit was provided by this treatment. These included a reduction in the requirement for laser retinopathy (5.2% vs. 3.6%, for a 30% reduction; *P *= 0.0003) and a reduction in albuminuria (2.5% absolute reduction and 1.2% regression; *P *= 0.002).

The FIELD study design allowed for statin therapy or other lipid-lowering drugs to be added at any time after randomization to either the fenofibrate arm or the placebo arm. The average use of other lipid-lowering therapies (mainly statins) was 17% in the placebo patients and 8% in the fenofibrate patients (*P *< 0.0001). Significant differences existed also in the use of other in-treatment therapies between the two treatment arms, including angiotensin- converting enzyme (ACE) inhibitors (*P *= 0.003), beta-blockers (*P *= 0.01), diuretics (*P *= 0.006), and coronary revascularization procedures (*P *= 0.003), with the greater use always occurring in placebo patients. There was a continual increase in statin use through the course of the study, and by the end of the study the statin drop-in rate was 36% in the placebo patients and 19% in the fenofibrate patients. Initiation of statin therapy and other secondary preventive therapies such as aspirin, ACE inhibitors, and beta-blockers also occurred at higher rates in patients with a prior history of CAD compared with patients with no prior history of CAD. The differential use of statins and other evidence-based therapies significantly attenuated the benefits of fenofibrate therapy. Adjustment for statin use revealed a pronounced reduction of total cardiovascular events.

A second explanation for the negative outcome of FIELD related to the change in lipids with fenofibrate, which was considerably less than expected for HDL cholesterol: it was increased by just 5% (compare, for example, with 18% increasing of HDL cholesterol by bezafibrate in the Bezafibrate Infarction Prevention (BIP) trial)).

#### Bezafibrate: emerged benefits in metabolic syndrome

Bezafibrate, in comparison with other fibrates, has an unique characteristic profile of action since it activates all three PPAR subtypes (alpha, gamma and delta) at comparable doses [25-27]. Therefore, bezafibrate operates as a pan-agonist for all three PPAR isoforms. In two old studies bezafibrate decreased the rate of progression of coronary atherosclerosis and decreased coronary event rate [15,16]. In another large trial in 1568 men with lower extremity arterial disease, bezafibrate reduced the severity of intermittent claudication [17]. In general, the incidence of coronary heart disease in patients on bezafibrate has tended to be lower, but this tendency did not reach statistical significance. However, bezafibrate had significantly reduced the incidence of non-fatal coronary events, particularly in those aged <65 years at entry, in whom all coronary events may also be reduced. In the BIP study an overall trend of a 9.4% reduction of the incidence of primary end point (fatal or non-fatal myocardial infarction or sudden death) was observed. The reduction in the primary end point in 459 patients with high baseline triglycerides (200 mg/dL) was significant [18].

Most recent extensions of the BIP trial give further support to the hypothesis that patients with insulin-resistant syndromes such as diabetes or metabolic syndrome might be the ones to derive the most benefit from therapy with fibrates [20,28-30]. Bezafibrate can reduce the incidence of MI in patients with metabolic syndrome. Overall, bezafibrate treatment was associated with reduced risk of any MI and non-fatal MI with HR (CI) respectively 0.71 (0.54–0.95) and 0.67 (0.49–0.91). The cardiac mortality risk tended to be lower on bezafibrate (HR 0.74, CI 0.54–1.03). This trend persisted in patients with augmented features of metabolic syndrome(at least 4 risk factors for metabolic syndrome); of note, a marked reduction in cardiac mortality was observed among these patients on bezafibrate (HR 0.44, CI 0.25–0.80).

Measurements obtained during placebo treatment within BIP trial demonstrated a natural history of progressive increasing of insulin resistance over long-term follow-up [28]). These unfavorable longitudinal changes were stopped when patients used bezafibrate. In addition, reduced incidence of type 2 diabetes in patients on bezafibrate has been demonstrated [29,30]. These new data raise the intriguing possibility that bezafibrate and other fibrates may eventually prove to be clinically useful for conditions other than dyslipidemia [31].

The factor that dominates in overweight-related metabolic syndrome is the permanent elevation of plasma free fatty acids (FFA) and the predominant utilization of lipids by the muscle inducing a diminution of glucose uptake and insulin resistance. Currently, an insulin-resistant state – as the key phase of metabolic syndrome – constitutes the major risk factor for development of macrovascular complications [32-35].

On the basis of the current concept of the evolution of adipogenesis via PPAR modulation toward insulin resistance and atherothrombotic macrovascular complications (including MI), the decreasing of plasma FFA and improving of insulin sensitization by PPAR agonists seems to be a logical and valuable goal for therapy.

It is important to note that on a whole-body level, lipid and glucose metabolisms interact intimately. Briefly, PPAR alpha is activated by fibric acids (e.g. bezafibrate) and form heterodimers with the 9-cis retinoic acid receptor (RXR). These heterodimers bind to peroxisome proliferator response elements, which are located in numerous gene promoters and increase the level of the expression of mRNAs encoded by PPAR alpha target genes. Bezafibrate reduces triglyceride plasma levels through increases in the expression of genes involved in fatty acid-beta oxidation and by decrease in apolipoprotein C-III gene expression. Fibric acids increase HDL-C partly by increasing apolipoprotein A-I and apolipoprotein A-II gene expression. Their triglyceride-lowering and HDL-C raising effects lead to decreased systemic availability of fatty acid, diminished fatty acid uptake by muscle with improvement of insulin sensitization and reduced plasma glucose level [36-39].

Evidence also suggests that there is a 'fibrate effect' that mediates the reduction in CAD risk beyond the favorable impact of these agents on HDL-C levels. This last notion is consistent with the pleiotropic effects of fibrates which are known to be related to their mechanisms of action [40]. Through PPAR alpha, fibrates have a significant impact on the synthesis of several apolipoproteins (apo) and enzymes of lipoprotein metabolism as well as on the expression of several genes involved in fibrinolysis and inflammation. Such changes contribute to improve the catabolism of triglyceride-rich lipoproteins, leading to a substantial increase in HDL-C levels accompanied by a shift in the size and density of LDL particles: from small, dense LDL particles to larger, more buoyant cholesteryl ester-rich LDL. These observations becomes particularly important given the dramatic increase in obesity, diabetes, and metabolic syndrome, conditions associated with low HDL and high triglyceride levels and small, dense LDL particles, the lipid profile for which fibrates would seem to be ideally suited [31].

However, different fibrates may have a somewhat different spectrum of effects. Pooled together evidence suggests, that gemfibrozil and bezafibrate have optimum cardiovascular benefit in metabolic syndrome and/or other appearances of insulin resistance.

### Nicotinic acid

Nicotinic acid (or niacin) has beneficial effects on all traditional blood lipid and lipoprotein fractions, particularly for increasing high-density lipoprotein (HDL)-cholesterol and reducing lipoprotein(a). Nicotinic acid has been used for the treatment of dyslipidemia since the 1950's, but the mechanism of action has only recently been elucidated. Niacin, a vitamin of the B complex which participates in tissue respiration oxidation-reduction reactions, decreases the fractional catabolic rate of apoA-I *via *reduction in hepatocytes uptake [41]. Increasing apoA-I would facilitate greater RCT by making apoA-I more bioavailable to remove excess cellular cholesterol from the arterial wall macrophage. Niacin also inhibits hepatic diacylglycerol acyltransferase 2 (DGAT2) which is a key enzyme in the synthesis of triglycerides destined for VLDL [42]. Nicotinic acid additionally inhibits adipose tissue lipolysis by inhibiting hormone-sensitive triglyceride lipase [43]. It is through this combination of action that nicotinic acid exerts its changes upon lipid parameters – increased HDL, lowered LDL and TG – and the clinical consequences of these effects have been positively borne out in clinical trials.

The benefits of niacin therapy upon cardiovascular events and mortality was first demonstrated in the Coronary Drug Project (CDP), a randomized, double-blind, placebo-controlled trial on 8,341 men with prior myocardial infarction that was started in 1966 [44]. Significantly fewer cardiovascular events and a mortality benefit were seen at the conclusion of the original trial after 6 years of follow-up, and these results persisted 15 years after the initiation of niacin [45]. Niacin therapy, however, is poorly tolerated by patients primarily because of skin flushing. Of subjects taking immediate-release niacin 85% experience flushing [46]; in fact, 75% of patients in the niacin arm of the CDP dropped out of the study [47]. The flushing issue has been ameliorated by the introduction of slow-release niacins – flushing for these products is approximately 26% [46] – and pre-medication with aspirin. However, slow-release niacins lead to hepatotoxicity, which appear to be caused by metabolites of the nicotinamide metabolic pathway.

New prolonged-release nicotinic acid designed to produce less vasodilatory flushing than crystalline immediate-release nicotinic acid and less hepatotoxicity than previous sustained-release formulations of nicotinic acid [48]. Despite the benefit of this therapy, patient adherence is poor. Nicotinic acid has also been criticized for dysregulation of glycemic control [49]: nicotinic acid therapy, particularly in large doses, can decrease insulin sensitivity and increase plasma glucose levels [50,51]. Probably, this effect for prolonged-release nicotinic acid is less than previously reported for crystalline nicotinic acid [52].

### Bile acid sequestrants

There are three most popular bile acid sequestrants: cholestyramine, colesevelam, and colestipol. These drugs' principal mechanism of action is the binding of bile acids within the intestinal lumen thereby reducing the reabsorption of bile acids and available intrahepatic cholesterol. Partial diversion of the enterohepatic circulation using bile acid sequestrants depletes the endogenous bile acid pool by approximately 40%, thus stimulating an increase in bile acid synthesis from cholesterol, which lowers low-density lipoprotein cholesterol by 15 to 26%. The mechanism by which HDL is raised is through increased intestinal production of apoA-I [53]. The largest trial to study a bile acid sequestrant as monotherapy for hypercholesterolemia was the Lipid Research Clinics Coronary Primary Prevention Trial. This trial of 3,806 hypercholesterolemic men without CAD found a 19 percent reduction in the incidence of CAD in the men treated with cholestyramine [54]. Bile acid sequestrants are not absorbed by the intestine and thus have no systemic drug-drug interactions, but may interfere with the absorption of some drugs [55]. The use of bile acid sequestrants is limited by patient adherence as these drugs commonly cause gastrointestinal side effects, especially constipation, and require large and frequent dosing. The effect on HDL elevation is usually negligible. Lastly, for the dyslipidemic patient who concomitantly has high triglycerides, these drugs have no beneficial effect.

### Ezetimibe

Ezetimibe is a novel cholesterol absorption inhibitor that blocks the translocation of dietary and biliary cholesterol from the gastrointestinal lumen into the intracellular space of jejunal enterocytes [56]. Similar to the bile acid sequestrants, ezetimibe reduces intestinal cholesterol absorption by binding to the apical cholesterol export pumps ABC proteins. The ABC transporters are located in the intestinal enterocytes brush border and promote efflux of dietary cholesterol and plant sterols from enterocytes back into the intestinal lumen, thus limiting the amount of absorbed cholesterol [57]. Experimental models suggest that ezetimibe, similar to other lipid-modifying agents, results in reduced atherosclerosis; in apoE-knockout mice, ezetimibe administration resulted in reduced carotid and aortic atherosclerotic development [58]. The coadministration of ezetimibe with a statin has yet to be proven to have a morbidity or mortality advantage over uptitration of statin monotherapy. Overall, ezetimibe has a favourable drug-drug interaction profile, as evidenced by the lack of clinically relevant interactions between ezetimibe and a variety of drugs commonly used in patients with hypercholesterolaemia. Ezetimibe does not have significant effects on plasma levels of statins, fibrates, digoxin, glipizide, warfarin and triphasic oral contraceptives. Higher ezetimibe exposures were observed in patients receiving concomitant ciclosporin, and ezetimibe caused a small but statistically significant effect on plasma levels of ciclosporin. Because treatment experience in patients receiving ciclosporin is limited, physicians are advised to exercise caution when initiating ezetimibe in the setting of ciclosporin coadministration, and to carefully monitor ciclosporin levels.

### CETP inhibition

Increasing HDL-cholesterol levels by pharmacological inhibition of the CETP is currently under intense investigation. Two small-molecule compounds, JTT-705 and torcetrapib, have been shown to effectively increase HDL-c levels in humans, without inducing clinically significant side effects when used as monotherapy or combined with statins [59,60]. Whether this approach will translate into a reduction in risk of atherosclerotic disease has not yet been established. Elevated HDL secondary to CETP deficiency may not then be entirely atheroprotective. An inhibition of CETP would allow the accumulation of lipid-laden HDL, but not lipid-deficient HDL. One hypothesis that could support the epidemiologic findings is that although HDL is increased, more atherogenic low density particles could accumulate, and if HDL is already lipid-laden, there would be less lipid-deficient HDL to participate in RCT. Animal models suggest, however, that partial inhibition of CETP results in reduced atherosclerosis. Clinical trials of CETP inhibitors in humans have resulted in impressive increases in HDL. Torcetrapib, a small molecule inhibitor of CETP, increased HDL by 46 to 106% without significant change in other lipid parameters [61]. Another small molecule inhibitor JTT-705 increased HDL by 34% with a modest 7% decrease in LDL [62]. Clinical trials with hard cardiovascular endpoints are pending, and an intravascular ultrasound (IVUS)-based imaging endpoint study of torcetrapib is also underway.

### Combined therapy: approaches to optimization lipid-lowering managment

Whereas statins remain the drug of choice for patients who need to achieve the LDL-C goal, fibrates, niacin or bile acid sequestrants may represent the alternative intervention for subjects with atherogenic dyslipidemia typical for metabolic syndrome and an LDL-C already close to goal values. In addition, the concomitant use of fibrates or niacin seems to be attractive in patients whose LDL-cholesterol is controlled by statin therapy but whose HDL-cholesterol and/or triglyceride levels are still inappropriate [63-67]. This strategy will be tested in the ongoing Action to Control Cardiovascular Risk in Diabetes (ACCORD) trial [68]. Although like FIELD, fenofibrate is used in ACCORD to treat diabetes, unlike the FIELD study fenofibrate is not being used as monotherapy but only in combination with simvastatin to compare with simvastatin therapy alone. This design should largely avoid the problem of off-trial drug use encountered in FIELD and at the same time might solidify a role for fenofibrate as a specific adjunct to statin therapy in the treatment of diabetic dyslipidemia [68].

## Conclusion

Controlled clinical trials show similar or even greater cardiovascular benefits from statins-based therapy in patient subgroups with diabetes, impaired fasting glucose, and metabolic syndrome, compared with overall study populations [69]. Therefore, statins are the drug of first choice for aggressive lipid lowering actions and reducing risk of coronary artery disease in these patients with combined atherogenic dyslipidemia. However, current therapeutic use of statins as monotherapy is still leaving many patients with mixed atherogenic dyslipidemia at high risk for coronary events and commonly insufficient to achieve all lipid targets recommended by current guidelines. For this reason, other approaches to treatment of combined hyperlipidemia should be considered. Because fibrates, niacin, ezetimibe and statins each regulate serum lipids by different mechanisms, combination therapy may offer particularly desirable benefits in patients with combined hyperlipidemia.

A combination statin/fibrate or statin/niacin therapy may be often necessary to control all lipid abnormalities in patients with metabolic syndrome and diabetes adequately, since fibrates and niacin provide additional important benefits, particularly on triglyceride and HDL-cholesterol levels. Thus, this combined therapy concentrates on all the components of the mixed dyslipidemia that often occurs in persons with diabetes or metabolic syndrome, and may be expected to reduce cardiovascular morbidity and mortality.

Safety concerns about some fibrates such as gemfibrozil may lead to exaggerate precautions regarding fibrate administration and therefore diminish the use of these agents. However, other fibrates (such as bezafibrate and fenofibrate) appear to be safer and better tolerated [70-76]. In addition, bezafibrate as pan-PPAR-activator has clearly demonstrated beneficial pleiotropic effects related to glucose metabolism and insulin sensitivity. Therefore a proper co-administration of statins with other agents: fibrates, niacin [77] or ezetimibe [78] – selected on basis of their safety and effectiveness, could be more valuable in achieving a comprehensive lipid control as compared with statins monotherapy.

## Abbreviations

ACE – angiotensin converting enzyme

ACCORD – Action to Control Cardiovascular Risk in Diabetes

ATP – Adult Treatment Panel

BIP – Bezafibrate Infraction Prevention

CAD – coronary artery disease

CETP – cholesteryl ester transfer protein

DM – diabetes mellitus

FIELD – Fenofibrate Intervention and Event Lowering in Diabetes

FFA – free fatty acids

HDL – high-density lipoprotein

HHS – Helsinki Heart Study

LDL – low-density lipoprotein

MI – myocardial infarction

NCEP – National Cholesterol Education Program

PPAR – peroxisome proliferator activated receptor

VA-HIT – Veterans Affairs High-density lipoprotein cholesterol Intervention Trial

## Competing interests

The author(s) declare that they have no competing interests.

## Authors' contributions

All authors have equally contributed in the conception and drafting of the manuscript.
